# Serologic Evidence of Leptospirosis in Humans, Union of the Comoros, 2011

**DOI:** 10.3201/eid2004.131207

**Published:** 2014-04

**Authors:** Yann Gomard, Rahamatou Silai, Géraldine Hoarau, Ketty Bon, Florelle Gonneau, Amina Yssouf, Alain Michault, Koussay Dellagi, Pablo Tortosa

**Affiliations:** Centre de Recherche et de Veille sur les Maladies Emergentes dans l’Océan Indien (CRVOI), Ste. Clotilde, La Réunion, France (Y. Gomard, K. Dellagi, P. Tortosa);; Programme National de Lutte contre le Paludisme (PNLP), Moroni, Comoros (R. Silai, A. Yssouf);; Centre Hospitalier Universitaire, St. Pierre, La Réunion (G. Hoarau, K. Bon, F. Gonneau, A. Michault);; Université de La Réunion, Ste. Clotilde (Y. Gomard, P. Tortosa);; Unité de Recherche sur les Maladies Infectieuses et Tropicales Emergentes, Marseille, France (A. Yssouf);; Institut de Recherche pour le Développement, Ste Clotilde (K. Dellagi)

**Keywords:** leptospirosis, humans, Union of the Comoros, zoonoses, Leptospira, bacteria, tropical islands, microscopic agglutination test

**To the Editor:** Leptospirosis is a worldwide bacterial zoonosis caused by infection with pathogenic *Leptospira* spp. (Spirochaetales, Leptospiraceae). Most mammals can be infected, but rats are considered the main reservoir, maintaining *Leptospira* spirochetes in the lumen of renal tubules and contaminating the environment with bacteria-infected urine. Transmission to humans is accidental, occurring through contact with animal secretions or with contaminated environmental materials.

In temperate countries, human leptospirosis is a sporadic disease; incidence is much higher in the tropics because climate and environmental conditions are conducive to the survival of bacteria, resulting in increased exposure of humans to leptospirosis-causing pathogens (*1*). Among islands in the southwestern Indian Ocean, human leptospirosis is endemic to Mayotte, France, and La Réunion (*2*–*4*) and to the Seychelles, where the incidence of leptospirosis is one of the highest worldwide (*5*). Leptospirosis is poorly documented in other islands in the region, including Mauritius, Madagascar, and the Union of the Comoros (*2*,*6*–*8*). Whether the scant documentation indicates underdiagnosis or reflects local epidemiologic specificities is unknown. To improve knowledge of *Leptospira* infection in the region, we conducted a study in the Union of the Comoros to serologically assess the presence or absence of leptospirosis in humans. The Union of the Comoros consists of 3 islands: Grande-Comore, Mohéli, and Anjouan. Together with a fourth, southern island, Mayotte, these islands form the Comoros Archipelago. 

For feasibility reasons, we used excess serum samples. Seventy-six samples were from healthy volunteers who gave informed consent; 318 clinical blood samples from patients had been obtained by private laboratories and by the surveillance laboratory of the National Malaria Control Programme (PNLP) during August 1–October 8, 2011. The Ministère de la Santé, de la Solidarité et de la Promotion du Genre of the Union of the Comoros, authorized the serologic investigation (authorization no. 1175/MSSPG/DNS).

We used the microscopic agglutination test (MAT) to test serum samples; the MAT was based on a panel of 15 *Leptospira* strains, enabling the screening of all recently reported serogroups for human and animal cases on neighboring Mayotte (*2*,*4**,**9*). A list of the tested strains follows, shown as *Genus species* Serogroup/Serovar (type strain): *L. borgpetersenii* Ballum/Castellonis (Castellon 3), *L. borgpetersenii* Sejroe/Hardjobovis (Sponselee), *L. borgpetersenii* Sejroe/Sejroe (M 84), *L. borgpetersenii* Tarassovi/Tarassovi (Perepelicin), *L. interrogans* Australis/Australis (Ballico), *L. interrogans* Autumnalis/Autumnalis (Akiyami A), *L. interrogans* Bataviae/Bataviae (Van Tienen), *L. interrogans* Canicola/Canicola (Hond Utrecht IV), *L. interrogans* Hebdomadis/Hebdomadis (Hebdomadis), *L. interrogans* Icterohaemorrhagiae/Copenhageni (Wijnberg), *L. interrogans* Pyrogenes/Pyrogenes (Salinem), *L. kirschneri* Cynopteri/Cynopteri (3522C), *L. kirschneri* Grippotyphosa/Grippotyphosa (Moskva V), *L. kirschneri* Mini/Undetermined serovar (200803703) (*9*), *L. noguchii* Panama/Panama (CZ214K). Each serum sample was tested at dilutions ranging from 1:50 to 1:3,200 and considered positive when the MAT titer was >100.

Our serologic findings showed evidence of *Leptospira* infection in humans on the 3 islands of the Union of the Comoros (MAT titers 100–1,600, geometric mean titer [GMT] 194). The positivity rate was 10.3% (95% CI 4.8–15.9) for samples from Mohéli, 4.2% (95% CI 1.4–7.0) for samples from Grande-Comore, and 3.4% (95% CI 0.1–6.7) for samples from Anjouan; no significant difference was found between islands or by the age or sex of residents (p>0.05, Fisher exact test). *Leptospira* infection was more prevalent and MAT titers were higher among serum samples from the patient group than the healthy donor group (20 positive samples/318 total vs. 3 positive samples/76 total; GMT 207 vs. GMT 126), but the difference was not significant (p>0.05, Fisher exact test). In 78% of seropositive serum samples, antibodies reacted with serogroups Australis, Bataviae, Grippotyphosa, Panama, Pomona, Pyrogenes, Mini, and/or Sejroe. MAT titers >100, which are suggestive of more specific antibodies to *Leptospira*, were observed for all serogroups except Australis and Sejroe. Pyrogenes serogroup was identified in one third of positive samples from Mohéli and was associated with the highest agglutination titers (Figure).

**Figure:**
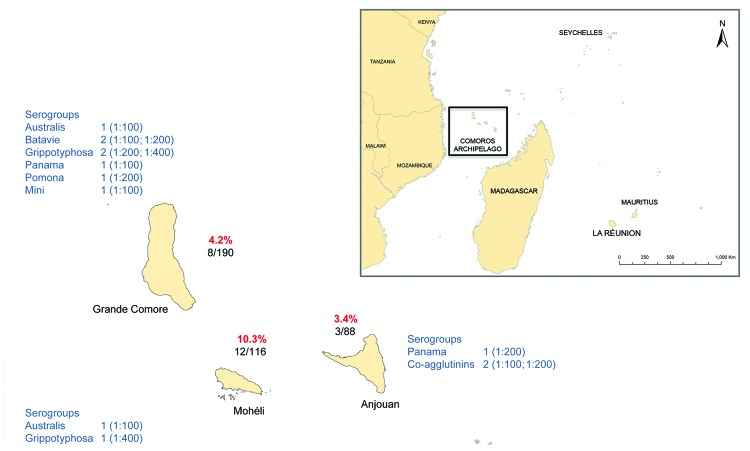
Microagglutination test results, showing serologic evidence of leptospirosis in humans, Union of the Comoros, 2011. The percentage of positive cases is shown for each island; the number below the percentage indicates the number of positive serum samples/total number tested. The serogroups identified on each island are shown; numbers represent the number of positive serum samples and, in parentheses, the number of corresponding titers. When agglutination was observed with >1 serogroup, the serogroup with a titer difference >2 relative to other serogroups was considered to be the infecting serogroup; when no serogroup had a titer difference >2 relative to other serogroups, coagglutinins were considered to be present in the serum sample. Data for Mayotte Island are from previous studies (*2**,**4*).

Our data indicate that *Leptospira* infections do occur in humans in the Union of the Comoros; this finding is consistent with those in studies reporting leptospirosis in persons returning from travel in the Union of the Comoros (*2*,*8*) and with the detection of pathogenic *Leptospira* spp. in bats sampled on these islands (*10*). The human leptospirosis–related serologic findings in Union of Comoros are most comparable to those from neighboring Mayotte, where leptospirosis is mainly caused by serogroups Mini/Sejroe/Hebdomadis complex, Pyrogenes, Grippotyphosa, and Pomona and where serogroup Icterohaemorraghiae is not detectable (*2*). These findings contrast with human leptospirosis findings from La Réunion and the Seychelles, where the Icterohaemorraghiae serogroup is most common (*3*).

Our MAT-derived data cannot discriminate between recent and past *Leptospira* infections, nor can these data be used to determine the severity of the disease in the Union of the Comoros. Nonetheless, the data strongly support the presence of human leptospirosis on the 3 islands of the Union of the Comoros and emphasize the need for a proper diagnosis to ascertain the number of leptospirosis cases among the acute febrile illnesses in this country.
